# The New (or Old) Anæsthetic

**Published:** 1864-01

**Authors:** G. Q. Colton


[*Med. and Surg. Reporter.*

---

The New (or Old) Anæsthetic.—*By G. Q Colton.*—Being informed that you take an interest in the question as to whom the honor belongs of having discovered and made practical anæsthesia, I ask leave to bring to your notice some facts within my own knowledge, which would seem to conclude the whole matter.

No one, I think, who has taken pains to examine the evidence on the subject, can doubt that the honor of the discovery belongs to the late Dr. Horrace Wells, of Hartford, Conn. He made his discovery at one of my "exhibitions" of "nitrous oxide gas," in the city of Hartford, on the evening of the 10th of December, 1814, and the next day he brought that discovery to the test of an experiment on himself. I, at his request, administered to him the gas, and Dr. Briggs, a dentist, extracted one of his large molar teeth without the slightest pain. The success of the operation surprised us all. This was, no doubt, the first successful trial of an adequate anæsthetic agent. and led to all that has since followed in this beneficent line of effort.

He attempted to establish the truth and value of his discovery in Boston, soon after, calling on Dr. Jackson, Dr. Morton and many dentists of that city. These facts are established by an elaborate and unanimous report on the question "who discovered anæsthesia," made to the New York Legislature at the session of 1860, by the Medical Society of that State. This report contains an exhaustive consideration of the claims of Wells, Jackson and Morton, arriving at the conclusion that to Wells alone belongs the discovery. The facts are also established by many depositions, gathered and published in a volume on "anæsthesia," prepared by the Hon. Truman Smith. By these depositions Mr. Smith shows that Dr Wells was in the constant and successful use of nitrous oxide gas as an anæsthetic, in the city of Hartford, during nearly the whole of 1845. He occasionally used ether, but preferred the nitrous oxide for a brief operation, like the extraction of teeth. During this time the subject of anæsthesia had not obtained any hold on the public mind, except in the city of Hartford. The discoverer encountered almost insuperable incredulity. It could not be believed that *any* substance could be breathed so as to destroy pain in surgical operations! Surgeons and the medical profession treated the subject as chimerical. It is no wonder that Dr. Wells (a dentist) made very slow progress in the introduction of his new discovery.

In December, 1845, Dr. Wells went to Europe to bring out his new invention; during his absence Drs. Jackson and Morton commenced a series of experiments with ether, to determine what there was in this discovery; their experiments proved successful; their next step was to secure a patent, which they applied for in the month of *November*, 1846, nearly two years after Wells made and demonstrated his discovery. Before it was issued Jackson assigned his interest in the patent to Morton, therefore the patent was issued in the name of Morton. This, be it remembered, was all done in the absence of Wells. On the return of Wells to this country a discussion commenced between him and Morton as to the origin of the discovery, during which Wells died, leaving a widow in indigent circumstances with no means to prosecute the matter; consequently Morton had the field clear to tell his own story. By the disinterested and unpaid labors of the Hon. Truman Smith, the testimony was gathered which establishes beyond cavil the truth of the above statements.

At the time of the death of Wells, very few people, aside from eye-witnesses, had any faith in either nitrous oxide or ether as an anæsthetic, After this event the nitrous oxide was superceded by the use of ether, so far as any anæsthetic was used. Ether could be purchased at any drug-store, while it required expensive aparatus and some chemical knowledge to make the nitrous oxide; so that, for twenty years past, the nitrous oxide has hardly been thought of as an anæsthetic, till I revived it in May last, in New Haven, Conn. I there announced that I believed the nitrous oxide was a safe and efficient anæsthetic for the extraction of the teeth, and, on account of its harmlessness, was far superior to ether or chloroform. I arranged with Dr. Smith to extract teeth by this new (or old) anæsthetic; and in the space of three weeks and two days we extracted *over eight thousand teeth*, and without any pain to the patients. I then came to New York, and established the "Colton Dental Association," and have administered the gas, up to the present time, for the extraction of about eight thousand teeth. We find it works with far more certainty and uniformity than ether or chloroform; for we have never yet found a patient whom we could not put into a profound sleep. In no instance has any injurious effect attended the operation. We have given it to persons suffering with almost all sorts of disease, nervous debility and weakness and heart disease, including many cases of far-gone consumption, with no ill effects. In no instance have patients complained of pain. In a very few cases, where a large number of teeth have been extracted at a sitting, and several doses had to be given, a little blood would be swallowed, and nausea would result. With these rare exceptions, not the slightest unpleasant effects have followed.

We have never had a patient who had previously taken ether or chloroform that he did not warmly express his preference for nitrous oxide. Hundreds of dentists who were, at first, skeptical about the nitrous oxide *on seeing its opera-*

*tion*, have given it their warmest commendation; and I do not now know of *one* in New York, who does not prefer it to ether or chloroform. The opinion on this subject; so far as I have been able to learn, is universal; and a large number send us their patients requiring the extraction of teeth.

I think these facts ought to satisfy the medical, also the dental profession and public, that for a brief surgical operation, the nitrous oxide gas is the best anæsthetic known. I have no doubt that if desired, I could keep the patient in an anæsthetic state for fifteen minutes or more. Instead of producing anæsthesia by depressing the vital forces, and diminishing the circulation of the blood in fact, approaching the point of death, the gas produces anæsthesia from the very opposite cause, from *over-exhilaration*, and from an increased and more highly oxygenized condition of the blood. A small dose of nitrous oxide or ether or chloroform will exhilarate, a larger induces sleep. But ether and chloroform act directly on the nerves, and as a *stimulant.* A consequent reaction and depression follows. The nitrous oxide is not a stimulant, and does not act directly on the nerves at all. The gas is taken into the lungs aud is rapidly absorbed by the blood, producing a highly oxygenized condition of the fluid. This highly-purified blood acts as an *exhilarant* on the nerves. (There is a wide difference between an exhilarant and a stimulant.) The anæsthetia is produced by *over-exhilaration.* There is no reaction or depression following its inhalation. When I say this, I but reiterate what all works on chemistry state. I speak also from an experience of twenty years with the gas.

I call nitrous oxide a new anæsthetic, because I have after a long interval, revived the use of it. But it is in fact the old anæsthetic of Horace Wells; and I rejoice that Providence has permitted me to live and demonstrate the truth and validity of his great discovery. Surgeons, physicians and dentists from any part of the country, who may desire to verify the truth of the above representations, are invited to call and witness the operations at the rooms of the Colton Dental Association, 22 Bond street, New York.—*Med. and Surg. Reporter.*

